# Histology of the Nerves; a Contribution of the Latest Researches

**Published:** 1882-10

**Authors:** Frank Dudley Beane

**Affiliations:** New York City, Ex-Fellow of the Mass Med. Society; Ex-Member of the New York County Med. Society, etc.


### ARTICLE II.

### *Histology of the Nerves; a Contribution of the Latest Researches.**

BY FRANK DUDLEY BEANE, A. M., M. D., OF NEW YORK CITY,

Ex-Fellow of the Mass. Med. Society; Ex-Member of the New York County Med. Society, etc.

The recently-issued work of M. Ranvier contains a mine of wealth for those whose knowledge of the French language, and leisure permits them to explore the deeply-hidden and very interesting facts relative to the histology of the nerves.

To lay before the mass of the profession the salient and essential data of the latest and most reliable researches in an English dress is the object of the translator, whose belief in their value to the profession at large is his excuse for the publication of this paper.

*Cerebro-Spinal Nerves.*—The spinal and cerebral nerves proper are called, on account of their special constitution, *myelinic.* They are white, more or less opaque, emitting and reflecting light like watered silk. As Fontana † first pointed out, this pearly appearance is due to simple folds from which the light is reflected. If the nerve be stretched, these folds are seen to disappear. These folds are due to a zigzag arrangement of the nerve tube within the nerve when its sheath, by reason of its elasticity, has folded upon itself.

If a segment be removed from a nerve of a living animal (frog, rabbit, etc.) And allowed to rest in distilled water upon a glass slide, we see, after a certain time, masses of matter exude from the cut extremities, which possess the form of clews of transparent filaments. The latter gradu-

* Translated, abridge and arranged from "Lecons sur l' Histologie du Systeme Nerveux," par L. Ranvier, Professeur de l' Anatomie Generale au College de France. Paris. 1878.

† "Traite du Venin de la Vipere," t. ii, p. 202.

ally swell, their contour becomes less clear, they seem to dissolve one into the other, and, after half or one hour, the clews become divided into ball-like masses of varied form, double contour, highly refracting border, and concentric striæ, the latter somewhat reminding one of the original filaments; these masses free themselves from one another and float away to another portion of the microscopic field. They possess forms intermediate of the cylindrical and spherical. This medullary substance of nerve-tubes is called *myelin*, whence the designation of this kind of nerves.

If the nerve segment be undisturbed within the water, this process of oozing of the myelin would continue until a more or less extensive portion of the segment were completely freed of its medulla, leaving behind what, at first, appears to be simply a connective-tissue envelope. The *connective-tissue sheaths* of large nerve-trunks are three in number, from without inward:

*a.* The Perifascicular.

*b.* The Laminated.

*c.* The Intrafascicular.

A segment taken from a dog's sciatic nerve (which is composed of several fasciculi), and properly prepared * gives a specimen which beautifully shows the former two. The field shows the whole nerve as surrounded by an envelope of diffuse connective tissue, which also dips down and binds the various fasciculi together; this is the nerve's *perifasciculi sheath.*

Internal to the latter, and surrounding each fasciculus another envelope is seen, called the *laminated sheath.* Within, seemingly as offshoots from this sheath, appear delicate layers of connective-tissue which join each other here and there, forming, by their interspaces, compartments within the laminated sheath. These layers form what is called the *intrafascicular sheath.*

---

* Techanical details are necessarily omitted from this paper; the reader is referred to the original, where the minutest directions and best methods shall be found.—TRANSALATOR.

The compartments thereby formed, as well as the perifascicular sheath contain blood vessels.

Let us examine these various envelopes more in detail.

*Perifascicular Sheath.*—As mentioned above, this envelope surrounds the whole nerve as well as, by its prolongations, solders the different fascicula together. It is composed of diffuse connective-tissue, otherwise called cellular or areolar tissue. It is constituted by connective filaments, elastic fibres, flat cells, fat cells, blood vessels and lymphatics. The connective fibres, unlike those found elsewhere, do not intercross in all directions, but pursue a longitudinal course. The elastic tissue networks also present their elongated meshes in the direction of the nerve's axis. The fat cells are disposed in small elongated groups, the long diameter of which is parallel to the direction of the nerve-cord. The more internal portion of this sheath (*i. e.* nearest the nerve fasciculi) assume the form of laminæ, the filaments of which are, however, thick, large, and independent of each other. A properly prepared section shows their arrangement in the form of the leaves of an open book. These laminæ, instead of being composed of a trellis of delicate fibres like the laminated sheath, are, compared to the latter, only coarse mats. Indeed, it is impossible to draw a perfectly defined line between the connective-tissue of the perifascicular sheath and that constituting the laminated sheath proper. The distinction is arbitrarily based upon the fact that the filaments of the latter sheath are the more delicate. It is through the perifascicular sheath that the nerve is bound to the surrounding tissues.

*Laminated Sheath.*—This envelope, lying internal to the former, completely surrounds each fasciculus of nerve-tubes. A property prepared transverse section would show this sheath as composed of superimposed concentric layers.

If another section be prepared with silver nitrate, this sheath is seen to be constituted by a series of laminæ, each separated from the other by black, granular, often irregular lines, due to the staining material. The number of the laminæ depends upon the size of the nerve under examination; in this sheath of a single fasciculus, from a dog's sciatic nerve, ten to fourteen may be counted. In the rabbit they are fewer, more delicate, and so extensible that the injection separates them very fully.

The structure of these laminæ is made up of a *stroma* and *endothelial cells*, applied to the surface of the stroma. If a very delicate shred of this sheath be stripped off—so as to isolate, as nearly as possible, a single laminæ—and one or two drops of Bœhmer's solution of hœmatoxylon be allowed to fall upon it, if then it be washed in distilled water, and examined in glycerine, we shall see:

1. A slightly tinted (bluish) stroma of very delicate filaments, which decussate in such a manner as to form a network with irregular interstices.

2. Very deeply tinted nuclei, irregularly oval, variable in size, with a distinct nuclelous. There are in immediate contact with the stroma.

3. A homogeneous, transparent, delicate, slightly tinted layer, which unites the nuclei to each other.

The latter two form *endothelial layer* of the laminated sheath.

The layer, which connects the cells, is seen to present very delicate striæ which intersect each other at right-angles, and are less highly tinted than the nuclei proper. This layer is also thinner than the nuclei.

In man, the nuclei are so joined together (using the choroid membrane) that it is impossible to clearly distinguish the above-described characters; but if the chorid of a dog be used, we see them beautifully marked. Each face of the laminæ, which compose this sheath, is carpeted (so as to speak) by an endothelial layer. This fact can be well demonstrated in a specially-prepared Pacinian corpuscle; at times, by chance, we can see this arrangement in the laminated sheath of a nerve-fasciculus when properly prepared.

Between the different layers of this sheath are interspaces of irregular shape; these inter-lamellar interstices should be regarded as *serous* interspaces.

As M. Cruveilhier pointed out, in 1835, the laminated sheath should be considered a serous membrane, since, like all serous membranes, it is covered by endothelium. Besides the above constituents, this sheath contains, on the surface of each lamina, a species of network of delicate elastic fibres. A properly prepared specimen shows a species of plates with irregular contour, a central foramen, and the plates giving off fibrially prolongations from the periphery. These prolongations appear like a series of beads strung together as in a necklace. Similar grains or beads are sometimes irregularly distributed around the edge of the plate or its vicinity.

These plates, prolongations and grains are homologous in structure, powerfully refract light, are insoluble in acids and weak alkaline solutions, and are colored yellow by picric acid. The elastic tissue of this sheath appears in no other form than that just described, and it is extra—or pericellular, *i. e.*, developed external to the endothelial layer.

In the external laminæ of this sheath, round or oval openings are seen. They may appear as single foramina, or as one, two, three or more openings, lying side by side, with simple partitions between them.

The laminated sheath is pierced by arteries and veins of such size as to be visible to the naked eye, but no blood vessels are disturbed upon or within it.

*Intrafascicular Sheath.*—Between the nerve-tubes of each fasciculous the laminated sheath sends, from its most internal layers, partitions, which, dividing and subdividing, becoming thinner and thinner, divide the fasciculus into a great number of compartments. These partitions are the *intrafascicular lamina.* Finally, around each nerve-tube of the fasciculus. a very delicate sheath of connective tissue is found, and this membrane is called the *intrafascicular connective-tissue proper.* Whether this latter takes its origin from the former or from the internal layers of the laminated sheath, or whether it originates from neither, is not a settled question.

The intrafascicular connective-tissue *proper* is composed of fibres and cells.

If a nerve-cord be disassociated, (*i. e.*, nerve-tubes be separated from each other), after the action of acid osmic, we see that each nerve-tube is surrounded by a certain number of exceedingly delicate, slightly undulating fibrils, the direction of which is parallel to the nerve-tube. If another portion of this pre pared cord be stained by hœmatoxylon or picro carmine, we recognize cellular plates upon the surface of the fibrils. These cells are irregular in contour, and give off prolongations which do not join one cell to another, at least I have never seen them do so.

After detaching one of these cells, we find it does not possess a plane surface, but turns up its sides like a gutter-tile, thus preserving its normal shape around the nerve-tube. Some parts of its surface are thicker than others, its nuclei participating in this condition, which is due to depressions produced by the pressure of the neighboring connective-tissue fibres and of the nerve-tube itself.

The intrafascicular laminæ are supplied with blood vessels.

*Henle's Sheath.*—Descending the scale, following nerve-cords to a nerve of a single fasciculus, thence to a *funiculus* containing only two, three, or five individual nerve-tubes, we should find that this extremely small funiculus is surrounded by its own sheath, distinct from those already described.

This special sheath should be considered simply as the substitute for the laminated and intrafascicular sheaths, which are lost before the division of the fasciculus into these small funiculi. This sheath, which also envelops nerves composed of even *a single nerve-tube*, is the *sheath of Henle*, named after its discoverer.*

A segment of a lizard's (preferable) or frog's muscular nerve at its peripheral termination, where it is composed of a single nerve-tube, placed under the microscope, shows it to be surrounded by an external membrane, beneath which are nuclei imbedded in a mass of protoplasm. The membrane is pliant, as evidenced by the folds which are produced by the manipulation. It is carpeted on its internal surface (to which they adhere) by the above-mentioned nuclei, and, here and there on its external surface, we see the flat cells of its connective-tissue investment. A third species of cells are seen to cover the nerve-tube itself, beneath and absolutely distinct from the sheath of Henle.

---

* "Anatomie des Menschen;" edition of 1843.

If now a rat's or mouse's thoracic nerve be prepared with silver nitrate we see, beneath the connective-tissue which binds the nerve to the various external tissues, black lines which form large, irregular polygons, by their intervals. These lines correspond to spaces between the polygons, which latter are endothelial cells; the inter-polygonal spaces are filled up by an extremely delicate layer of intercellular cement. By carefully observing the specimen, we should see that there is a double *resean* of black lines which intercross each other, thus demonstrating there are two layers of endothelium which are closely applied to each other.

The membrane of Henle's sheath is composed of connective-tissue.

*En resume,* Henle's sheath is constituted by:

1. A connective-tissue membrane.
2. Lined internally by a layer of endothelial cells.
3. Internal to and distinct from the latter are nucleated cells which lie upon the nerve-funiculus or nerve-tube.

*Myelic Nerve-Tubes.*—Their structure presents a very interesting study to the histologist.

If a single nerve-tube be isolated, stripped of its sheath of Henle, and a segment be removed. placed upon a slide in a drop of distilled water, and examined under the microscope, the myelin is seen to comport itself in the same manner as described on page 254. The connective-tissue membrane seen at the end which has collapsed is the true envelope of the nerve-tube, first described by Schwann, and called after him, Schwann's sheath.

[TO BE CONTINUED.]

